# Supplementary material for: RAS-Beppu Classification: A New Recurrence Risk Classification System Incorporating the Beppu Score and RAS Status for Colorectal Liver Metastases
Source: Cancers (Basel). 2025 Feb 14;17(4):640. doi: 10.3390/cancers17040640 (PMC11853466; doi:10.3390/cancers17040640)
Supplement: Supplementary file 1 [file cancers-17-00640-s001.zip › supplementary tables.pdf]

cancers

MDPI

Supplementary Table S1. Patient characteristics based on the Beppu and RAS-Beppu classification.

	Beppu classification				*RAS*-Beppu classification			
	Low (n = 60)	Moderate (n = 61)	High (n = 52)	P value	Low (n = 43)	Moderate (n = 53)	High (n =77)	P value
Median age (range)	67 (25 - 94)	66 (27 - 85)	66 (37 - 84)	0.38	66 (25 - 94)	65 (27 - 85)	66 (37 - 85)	0.49
Gender (M/ F)	38/ 22	37/ 24	38/ 14	0.36	29/ 14	32/ 21	52/ 25	0.66
Median BMI (range)	22.6 (15 - 29)	22.8 15 - 31)	22.7 (16 - 33)	0.86	23 (16 - 29)	23 (15 - 31)	22 (15 - 33)	0.50
Primary tumor sight right/ left	16/ 44	14/ 47	10/ 42	0.65	10/ 33	13/ 40	17/ 60	0.95
Primary tumor T 0-2/ 3-4	8/ 50	6/ 54	5/ 47	0.74	5/ 36	8/ 44	6/ 71	0.39
Distribution of liver metastasis Unilobar/ Bilobar	53/ 7	44/ 17	21/ 30	<.0001	40/ 3	39/ 14	39/ 37	<.0001
Median CEA at diagnosis (range)	8.3 (0.9 – 281.7)	8.6 (0.5 – 961.4)	13.7 (1.9 – 53.5)	0.07	6.3 (0.9 – 281.7)	7.45 (0.5 – 961.4)	14.1 (0.9 - 3377)	0.047
RAS mutations (%)	17 (28)	25 (41)	22 (42)	0.23	0	17 (32)	47 (61)	<.0001
Median Beppu score (range)	4 (0 - 6)	8 (7 - 10)	14 (11 - 21)	<.0001	3 (0 - 6)	7 (0 - 10)	12 (7 - 21)	<.0001
Chemotherapy (%)								
Preoperative	10 (17)	26 (43)	36 (69)	<.0001	8 (19)	19 (36)	45 (58)	<.0001
Adjuvant	23 (38)	28 (46)	24 (47)	0.59	17 (40)	24 (45)	34 (45)	0.83
Operative procedures Non-AR/ AR	39/ 21	35/ 26	35/ 17	0.51	25/ 18	36/ 17	48/ 29	0.61
Laparoscopic surgery (%)	23 (38)	22 (37)	10 (19)	0.061	18 (42)	20 (38)	17 (22)	0.041
TSH or preoperative PVE (%)	2 (3)	2 (3)	1 (2)	0.88	2 (5)	1 (2)	2 (3)	0.71
Median operation Time (min) (range)	374 (90 - 718)	436 (205 - 1222)	473 (241 - 986)	0.0029	401 (90 - 718)	433 (116 - 1222)	456 (205 - 986)	0.057
Median intraoperative blood loss (ml) (range)	256 (0 - 1701)	380 (30 - 2089)	360 (10 - 4057)	0.29	280 (0 - 1701)	270 (15 - 2016)	360 (10 - 4057)	0.49
Transfusion of RBC (%)	2 (3)	5 (8)	5 (10)	0.38	1 (2)	4 (8)	7 (9)	0.37

Clavien-Dindo class ≧ III (%)	9 (15)	10 (16)	10 (19)	0.85	6 (14)	10 (19)	13 (17)	0.84
Resection status R0/ R1	49/ 6	39/ 10	33/ 10	0.23	34/ 5	43/ 4	44/ 17	0.021

BMI: body mass index, CEA: Carcinoembryonic Antigen, AR: anatomical resection, TSH: two-stage hepatectomy, PVE: portal vein embolization, RBC: red blood cells R1: resection margin positive, R0: resection margin negative.

Supplementary Table S2. Univariate analysis of disease-free survival and overall survival.

	DFS		OS	
	Univariate HR (95% CI)	P value	Univariate HR (95% CI)	P value
Age ≧ 70	0.93 (0.62 – 1.38)	0.71	1.35 (0.84 – 2.18)	0.22
Gender M/ F	1.14 (0.76 – 1.70)	0.52	0.91 (0.55 – 1.48)	0.70
BMI ≥ 22.5	0.92 (0.63 – 1.33)	0.66	0.88 (0.55 – 1.41)	0.61
Primary site: right/ left	1.32 (0.85 – 2.04)	0.21	1.72 (1.02 – 2.89)	0.041
Primary tumor: T 3-4/ 1-2	1.36 (0.66 – 2.8)	0.40	0.64 (0.32 – 1.29)	0.22
Bilobar/ Unilobar distribution	1.48 (1.00 – 2.19)	0.052	1.28 (0.79 – 2.09)	0.32
Preoperative CEA ≧ 100	1.25 (0.74 – 2.13)	0.41	1.61 (0.88 – 2.95)	0.13
RAS: mutant / wild	2.25 (1.54 – 3.29)	<.0001	2,44 (1.52 – 3.91)	0.0002
Beppu classification		.0009		0.066
Moderate / Low	1.83 (1.13 – 3.0)	0.014	1.36 (0.75 – 2.48)	0.31
High / Low	2.51 (1.55 – 4.07)	.0002	1.97 (1.11 – 3.50)	0.021
High / Moderate	1.37 (0.89 – 2.11)	0.155	1.45 (0.83 – 2.51)	0.19
RAS-Beppu classification		<.0001		0.0036
Moderate / Low	2.15 (1.19 – 3.90)	0.012	1.38 (0.67 – 2.85)	0.38
High / Low	3.50 (2.00 – 6.10)	<.0001	2.65 (1.39 – 5.07)	0.0031
High / Moderate	1.62 (1.06 – 2.48)	0.025	1.92 (1.11 – 3.32)	0.019
Preoperative chemotherapy	1.63 (1.12 – 2.38)	0.010	1.44 (0.83 – 2.52)	0.025
Adjuvant chemotherapy	0.79 (0.53 - 1.14)	0.19	0.73 (0.45 – 1.16)	0.18
Non-AR/ AR	1.26 (0.86 – 1.85)	0.24	1.46 (0.91 – 2.43)	0.11
Laparoscopic surgery	0.80 (0.53 – 1.21)	0.29	0.57 (0.32 – 1.00)	0.051
TSH or PVE	1.48 (0.36 – 6.05)	0.58	6.29 (1.23 – 32.3)	0.0051
Operating time ≧ 340	1.29 (0.84 – 1.99)	0.25	1.57 (0.89 – 2.77)	0.12
Intraoperative blood loss ≧ 210	1.31 (0.88 – 1.94)	0.18	1.77 (1.06 – 2.97)	0.03
Transfusion of RBC	0.93 (0.41 – 2.12)	0.86	1.33 (0.54 – 3.31)	0.54

Resection margin: R1 / R0	1.76 (1.05 – 2.96)	0.033	1.63 (0.86 – 3.10)	0.13
Clavien–Dindo ≧ III	1.00 (0.60 – 1.65)	0.99	2.1 (1.21 – 3.66)	0.012

BMI: body mass index, CEA: Carcinoembryonic Antigen, AR: anatomical resection, TSH: two-stage hepatectomy, PVE: portal vein embolization, RBC: red blood cells R1: resection margin positive, R0: resection margin negative.

Supplementary Table S3. Univariate and multivariate analysis of overall survival (OS) based on the Beppu and RAS-Beppu classification.

OS			Beppu classification		RAS-Beppu classification	
	Univariate HR (95% CI)	P value	Multivariate HR (95% CI)	P value	Multivariate HR (95% CI)	P value
Primary site: right/ left	1.72 (1.02 – 2.89)	0.041	1.78 (1.01 – 3.14)	0.045	1.91 (1.11 – 3.32)	0.020
RAS: mutant / wild	2,44 (1.52 – 3.91)	0.0002	2.81 (1.73 – 4.58)	<.0001		
Preoperative chemotherapy	1.71 (1.07 – 2.72)	0.025	1.44 (0.82 – 2.51)	0.20	1.24 (0.75 – 2.08)	0.40
TSH or PVE	8.12 (1.87 – 35.2)	0.0051	5.97 (1.16 – 30.8)	0.033	5.44 (1.04 – 28.5)	0.045
Intraoperative blood loss ≧ 210 (ml)	1.77 (1.06 – 2.97)	0.03	1.47 (0.85 – 2.54)	0.17	1.41 (0.82 – 2.43)	0.21
Clavien - Dindo ≧ III	1.97 (1.16 – 3.33)	0.012	1.85 (1.04 – 3.30)	0.036	1.79 (1.01 – 3.18)	0.046
Beppu classification		0.066		0.20		
Moderate / Low	1.36 (0.75 – 2.48)	0.31	1.26 (0.67 – 2.36)	0.47		
High / Low	1.97 (1.11 – 3.50)	0.021	1.86 (0.93 – 3.71)	0.078		
High / Moderate	1.45 (0.83 – 2.51)	0.19	1.47 (0.81 – 2.69)	0.21		
RAS-Beppu classification		0.0036				.0044
Moderate / Low	1.38 (0.67 – 2.85)	0.38			1.32 (0.62 – 2.80)	0.48
High / Low	2.65 (1.39 – 5.07)	0.0031			2.78 (1.37 – 5.67)	.0048
High / Moderate	1.92 (1.11 – 3.32)	0.019			2.12 (1.18 – 3.78)	0.011

HR: hazard ratio, TSH: two-stage hepatectomy, PVE: portal vein embolization. Beppu classification: Low: Beppu score ≦6, Moderate: 7 – 10, High: 11≦

Supplementary Table S4. Multivariate analysis for disease-free survival in the RAS-Beppu classification, modified clinical risk score (mCRS) and genetic and morphological evaluation (GAME) score considering RAS status.

DFS	*RAS*-Beppu classification		mCRS		GAME score	
	Multivariate HR (95% CI)	P value	Multivariate HR (95% CI)	P value	Multivariate HR (95% CI)	P value
Preoperative chemotherapy	1.02 (0.66 – 1.60)	0.92	1.23 (0.81 – 1.89)	0.33	1.18 (0.74 – 1.90)	0.49
Resection margin: R1 / R0	1.43 (0.83 – 2.45)	0.20	1.87 (1.10 – 3.19)	0.021	1.53 (0.90 – 2.63)	0.12
RAS-Beppu classification		.0002				
Moderate / Low	2.22 (1.17 – 4.22)	0.015				
High / Low	3.78 (2.00 – 7.12)	<.0001				
High / Moderate	1.70 (1.05 – 2.74)	0.030				
mCRS				.0023		
1/ 0			1.91 (1.00 – 3.65)	0.049		
2-3/ 0			3.12 (1.60 – 6.08)	.0008		
2-3/ 1			1.63 (1.05 – 2.55)	0.031		
GAME score						0.13
Medium/ Low					1.38 (0.77 – 2.47)	0.28
High/ Low					2.01 (1.01 – 3.98)	0.045
High/ Medium					1.46 (0.87 – 2.44)	0.16

R1: resection margin positive, R0: resection margin negative, mCRS: modified clinical risk score: total score corresponding to Node-positive primary tumor, largest liver metastasis >50 mm and RAS mutation in CRLM, expressed as 0-3 points. GAME score: genetic and morphological evaluation score: total points for the following 5 items; KRAS mutated tumors: 1 point, CEA ≥ 20 ng/mL: 1 point, Primary tumors lymph node metastasis: 1 point, 3 ≤ Tumor Burden Score (TBS) < 9: 1 point, TBS ≥ 9: 2 points extrahepatic disease: 2 points, Low: 0-1 points, Medium: 2-3 points, High: ≥ 4 points.

Supplementary figure legends:

Supplementary Figure S1: A treatment strategy based on the technical and oncological aspects of colorectal liver metastases.

Supplementary Figure S2: Disease-free survival based on the modified clinical risk score (mCRS) (a) and genetic and morphological evaluation (GAME) score classifications (b) (n=173).
