# Supplementary figures and images for: RAS-Beppu Classification: A New Recurrence Risk Classification System Incorporating the Beppu Score and RAS Status for Colorectal Liver Metastases
Source: Cancers (Basel). 2025 Feb 14;17(4):640. doi: 10.3390/cancers17040640 (PMC11853466; doi:10.3390/cancers17040640)

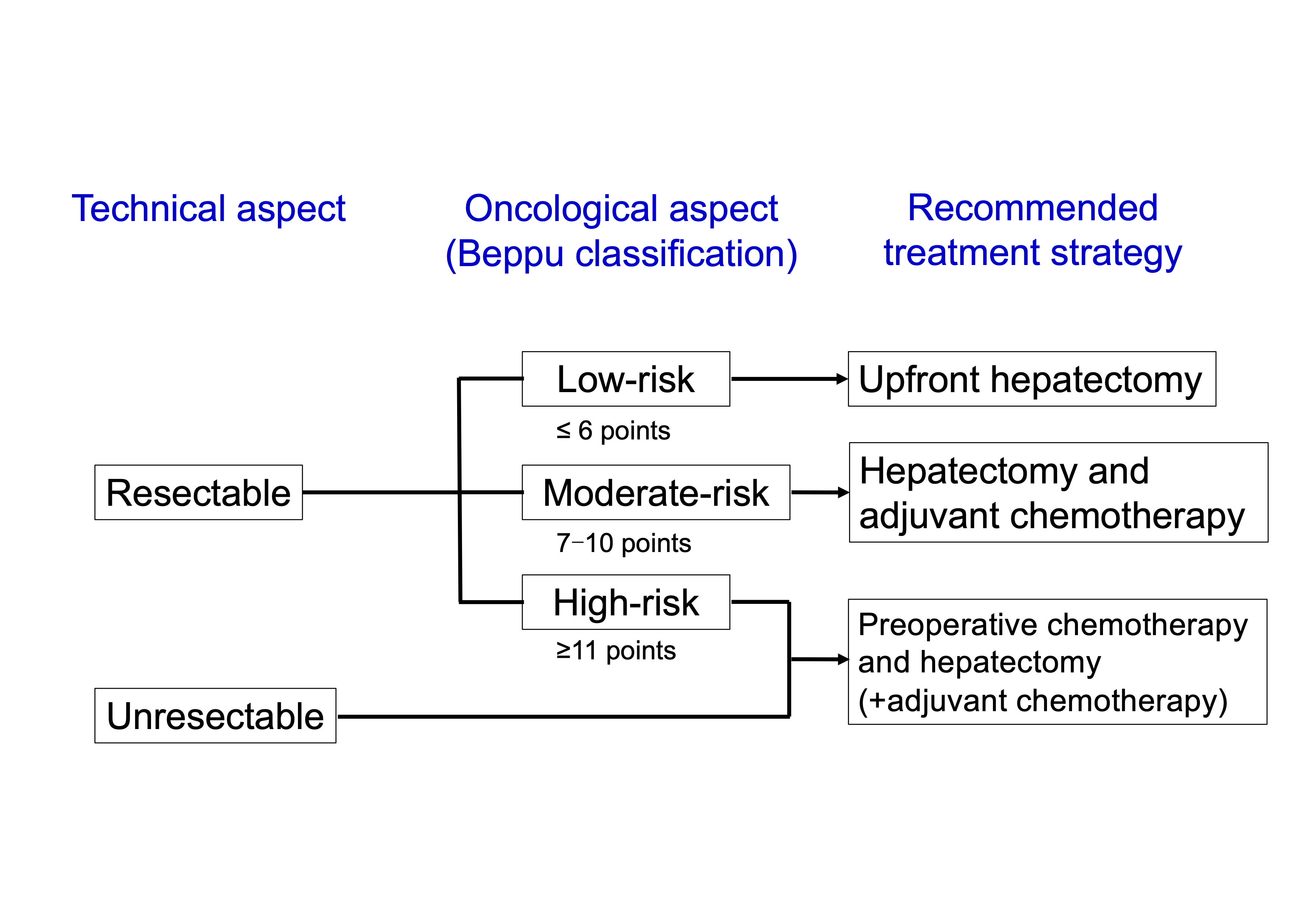

Supplement: Supplementary file 1 [file cancers-17-00640-s001.zip › supplementary figure S1.tiff]

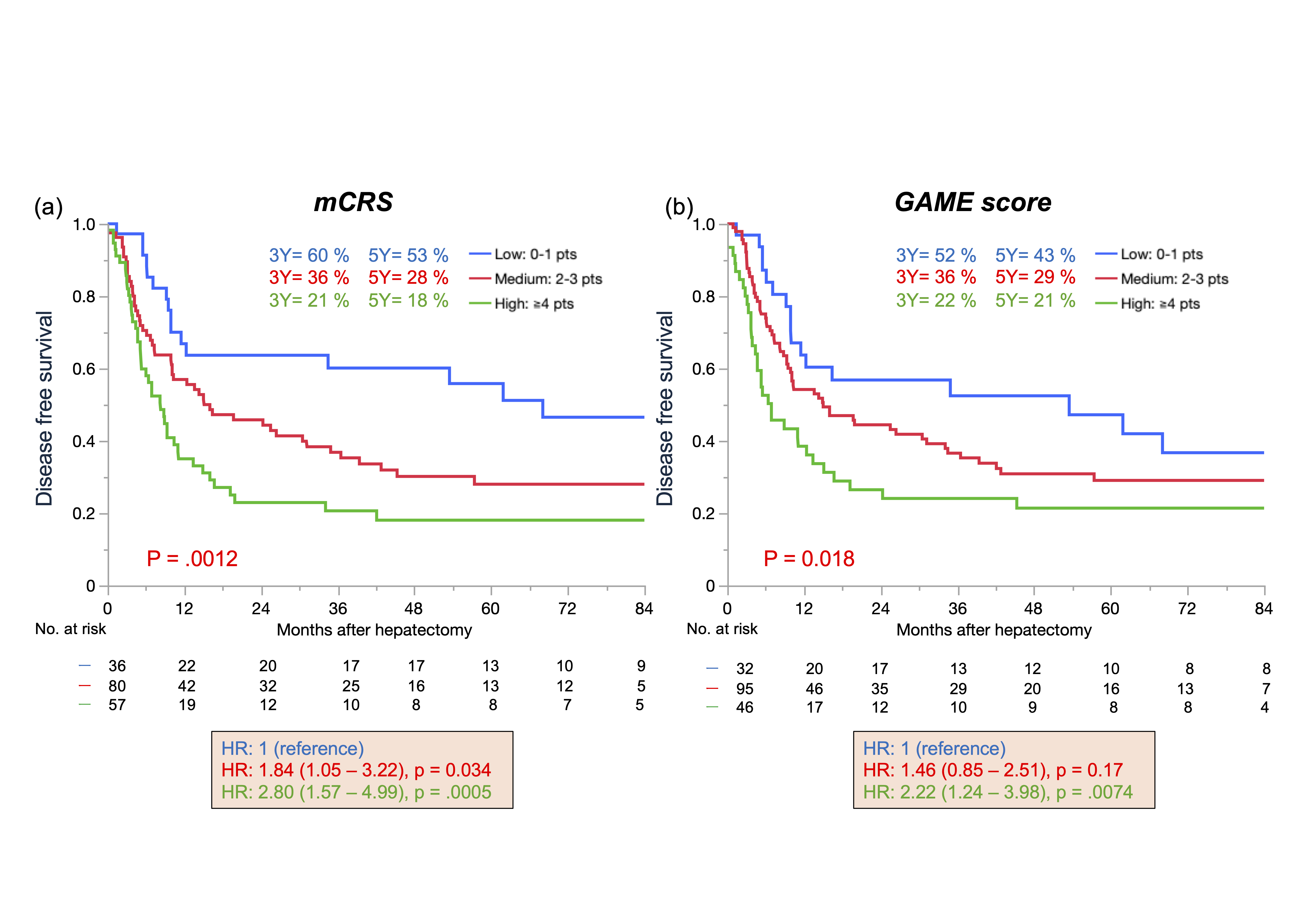

Supplement: Supplementary file 1 [file cancers-17-00640-s001.zip › supplementary figure S2.tiff]
